# Multiple nitrogen functionalized magnetic nanoparticles as an efficient adsorbent: synthesis, kinetics, isotherm and thermodynamic studies for the removal of rhodamine B from aqueous solution

**DOI:** 10.1038/s41598-019-45293-x

**Published:** 2019-07-04

**Authors:** Mike O. Ojemaye, Anthony I. Okoh

**Affiliations:** 1 SAMRC; Microbial Water Quality Monitoring Centre, University of Fort Hare, Alice, South Africa; 2 Applied and Environmental Microbiology Research Group (AEMREG), University of Fort Hare, Alice, South Africa

**Keywords:** Pollution remediation, Pollution remediation

## Abstract

The continuous demand for clean and affordable water needed for the survival of man is now a major challenge globally. Therefore, the treatment of wastewater generated from printing, textile and dyeing industries containing soluble dyes like rhodamine B (Rh-B) is of utmost important. This study investigates the efficiency of new multifunctionalized superparamagnetic nanoparticles (MNP-Tppy) for the removal of cationic Rh-B from aqueous solution. To afford MNP-Tppy, the surface of MNP was covalently functionalized with terpyridine ligand to enable an anionic charge on the adsorbent. The results of characterization including Brunauer-Emmett-Teller (BET) analysis, thermal gravimetric analysis (TGA), vibrating sample magnetometer (VSM), scanning electron microscope (SEM) and fourier transform infra–red spectroscopy (FTIR) indicate that this superparamagnetic nanoparticle functionalized with multiple nitrogen atoms was successfully synthesized. Adsorption experiments involving the effect of pH, time, temperature, adsorbent dose and adsorbate concentration show that the maximum adsorption of Rh-B using MNP-Tppy was observed at pH 9 and removal was observed to increase as solution pH increases. Similarly, time variation shows that adsorbate removal increases as adsorption time increases until the removal attained equilibrium at 15 min. Kinetic studies conducted among four kinetic models using the data obtained from effect of time indicate that the adsorption process can best be described by the pseudo-second order model. Isotherm studies conducted at three different temperatures revealed that Langmuir isotherm model fitted well for the equilibrium data with *q*_*m*_ value of 113.64 mg g^−1^ and thermodynamic studies showed that the adsorption process involving the removal of Rh-B from aqueous solution by MNP-Tppy is spontaneous, endothermic and realistic in nature. Lastly, Reusability experiments indicate that MNP-Tppy can be regenerated and re-used.

## Introduction

Recently, the heralding of multifunctional superparamagnetic nanomaterials with excellent chemical, physical, magnetic and biological characteristics has been a major topical issue in the field of science because of its importance in the conversion of energy^1,2^, medicine^3^, water treatment^4^, catalysis^5^ and so on. These materials with unique properties have been extensively studied to achieve an enhanced performance in the areas they are targeted or intended for. Magnetic nanoparticles as a member of a new advanced material have recorded tremendous successes in their application because of their use of specific site for attacking their target place, separation power brought about by magnetic means, high stability, large surface area, ability to tune their pore sizes and volumes. These properties qualify them as a great candidate for numerous applications because they are able to host different molecules with different functionalities, shapes and sizes. Be that as it may, most works on the use of these functional nanomaterials have focused on their application for sensors specifically oxygen sensing^6–8^, drug delivery^9–13^ but little works have been reported for multifunctional superparamagnetic nanoparticles as adsorbents for water treatment especially for dye removal from wastewater. Actually, different adsorbents including montmorrillonite, carbon nanotubes^14^, clay^15^, fly ash, activated carbon^16^ etc. have been employed for the removal of dyes from aqueous solution but encounters like inability to separate them from solution, generation of secondary pollutants, cost and unavailability are obvious challenges that limit their usage as adsorbents for the removal of dyes from wastewater.

Terpyridine ligands specifically 4-(2,2′:6′,2″-terpyridin-4′-yl)phenol are compounds with strong binding affinity and are currently attracting attentions in different areas of research including polymer science^17^, environmental science^18^, catalysis^19,20^, nanotechnology and medicine^21,22^. In nanotechnology, they can be employed for the covalent modification of inorganic nanomaterial for two reasons: (1) to stabilize the nanostructured material and (2) to functionalize the nanostructured material^23^. A typical terpyridine ligand contains three nitrogen atoms which are potent for the functionalization of MNPs by introducing multiple nitrogen moieties to the surface of MNPs.

Globally, fresh water supply has continued to decline as a result of global climate change, industrial growth and increasing population with this generating a great outcry from citizenry world over. Since fresh water resources are of limited supply, the recovery of wastewater or water contaminated with organics such as dyes is of utmost importance in order to ameliorate the sufferings experienced by man and animals from shortages in fresh water resources. Different strategies have been employed for the removal of contaminants from wastewater. Some of these include; photocatalysis^4^, coagulation/flocculation^24^, ion exchange^25^, reverse osmosis, membrane filtration^26^ and so on but challenges like the generation of sludge, operation difficulty, excessive energy consumption, cost burden and release of secondary pollutants into the environment are often encountered with these strategies which limit their usage. Adsorption due to its ease of operation, simplicity, low cost, reliability and effectiveness can overcome the challenges highlighted above and is recently been utilized for the removal of dyes from wastewater.

Cationic dyes such as rhodamine B (Rh-B) are widely used in the industry specifically textile, dyeing and printing companies. These dyes are positively charged and have different chemical structures with substituted aromatic groups. They are a basic dye which are water soluble and yield coloured ions in solution. Rhodamine B is a toxic substance and becomes very harmful to human and aquatic organisms upon the discharge of effluents contaminated with Rh-B to the environment^27^.

In this study, multiple nitrogen functionalized magnetic nanoparticles were synthesized and investigated for the removal of rhodamine B (Rh-B) from wastewater by adsorption. The novelty of this report is in the design and development of this new adsorbent; magnetic nanoparticles covalently functionalized with 4-(2,2′:6′,2″-terpyridin-4′-yl)phenol via its lateral substituent (hydroxyl group, -OH). This ligand possesses multiple nitrogen groups and has high chelating powers for the adsorption of rhodamine B from wastewater. To the best of our knowledge, no report has been published on the covalent functionalization of magnetic nanoparticles with terpyridine ligand, 4-(2,2′:6′,2″-terpyridin-4′-yl)phenol for the removal of rhodamine B from aqueous solution. Rhodamine B was considered in this study as a model dye for adsorption because of its extensive use in the industry, toxic nature and similarity in its behaviour and properties to other dyes in its class. Also, the mechanism and kinetics of adsorption of this dye necessary for practical and real life scenarios were studied from the adsorption processes involving effect of pH, contact time, temperature and initial adsorbate concentration and these serve as motivation for this work.

## Experimental

### Materials

Iron (II) chloride hexahydrate (Merck Chemicals), Iron (III) Chloride hexahydrate (Merck Chemicals), 2-acetylpyridine (Sigma Aldrich), 4-hydroxybenzaldehyde (Sigma Aldrich), tetraethoxysilane (Sigma Aldrich), 3-aminopropyltriethoxysilane (Sigma Aldrich), dicyclohexylcarbodiimide (Sigma Aldrich), 4-dimethylaminopyridine (SAAR Chem), dimethylformamide (BDH limited), Sodium hydroxide (Merck Chemicals), rhodamine B (Sigma Aldrich), 25% ammonia solution (Merck Chemicals), absolute ethanol (Merck Chemicals) were used for this study.

### Characterization

The textural and adsorptive properties of the synthesized materials were verified using a number of techniques. Fourier transform infrared spectrophotometer (FTIR) was used for the verification of the vibrations of the materials by placing a small sample on a Perkin Elmer Universal ATR sampling accessory spectrum 100 FT-IR spectrometer. For image collection, the materials were viewed using JOEL JSM-6390 scanning electron microscope; this was achieved by placing a small sample of the powder on a carbon double sided tape on a stub and coated with Au/Pd for a clearer image. The surface properties of the adsorbents were assessed using Micromeritics Tristar II 3020 surface area and porosity analyser (BET) with nitrogen flow gas. Magnetic measurements of the materials were taken with a VSM system of 14 T at a temperature range of 1.8 to 310 K. Thermal analysis of the MNPs samples were performed using a Perkin Elmer TGA 4000 analyzer under nitrogen at 20 mL min^−1^. The determination of point of zero charge (pHpzc) was carried out as we earlier reported in one of our published work^28^. This is important to enable an estimation of the electrical charge density of the adsorbent.

For 4-(2,2′:6′,2″-terpyridin-4′-yl)phenol (Tppy-OH) characterization, FTIR analysis was carried out. Also, Bruker AVANCE 400 MHz Nuclear Magnetic Resonance (NMR) spectrometer was used to obtain its ^1^H NMR and ^13^C spectra. Information on elemental composition of the ligand was obtained with the aid of a Vario-Elemental Microtube ELIII and Gallenhamp Melting Point apparatus gave information on the purity of the sample.

### Synthesis of 4-(2,2′:6′,2″-terpyridin-4′-yl)phenol (Tppy-OH)

**Figure 1:**
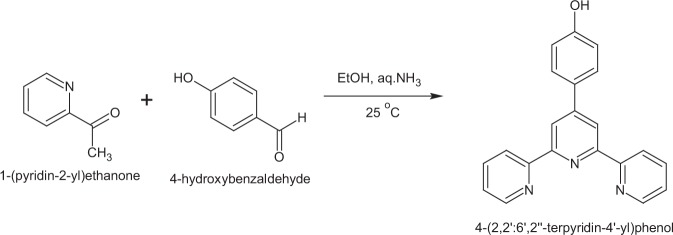
Synthesis of 4-(2,2′:6′,2″-terpyridin-4′-yl)phenol (Tppy-OH).

The synthesis of the ligand was achieved as described by Patel *et al*.^29^ with a slight modification. 1.211 g, 10.0 mmol of 2-acetylpyridine was added to a solution of 0.661 g, 5.0 mmol 4-hydroxybenzaldehyde previously dissolved in 20 mL of ethanol. A mixture of 0.013 mol NaOH solution and 15 mL aqueous ammonia solutions were added and left to stir overnight at room temperature (Fig. 1). The precipitate obtained was washed four times with 10 mL deionized water and two times with 10 mL absolute ethanol to yield an off white powder (615.5 mg, 55%). m.p. 198–200 °C; IR (ATR, cm^−1^) 3529, 3056, 1584, 1521, 1283; ^1^H NMR (400 MHz, DMSO-d_6_) δ: 8.80, 8.70, 8.05, 7.75, 7.80, 7.50, 6.95, 6.90, 2.5; ^13^C NMR (400 MHz, DMSO-d_6_) δ: 155.45, 155.20, 149.40, 149.20, 137.36, 128.09, 128.86, 120.80, 116.82, 116.52.

### Preparation of silica modified superparamagnetic nanoparticles

Silica modified magnetic nanoparticles were synthesized after the preparation of superparamagnetic nanoparticles. The superparamagnetic nanoparticles was synthesized according to the method previously reported by Maaz *et al*.^30^ by using iron (II) chloride hexahydrate and iron (III) Chloride hexahydrate in molar ration 1:2 under inert condition and basic pH. For the synthesis of silica modified magnetic nanoparticles, tetra ethyl ortho silicate (TEOS) was used to coat the surface of the MNPs and the details of this synthesis can be found in our recent report^28^.

### Preparation of amine functionalized superparamagnetic nanoparticles

Amine functionalized silica magnetic nanoparticles was prepared by treating silica magnetic nanoparticles with 3-aminopropyltriethoxysilane (APTES) according to a method reported in our previous study^31^. A small amount of APTES was added to silica magnetic nanoparticles (150 mg) previously dispersed in 30 mL absolute ethanol. This mixture was stirred under a reflux condenser at inert condition at 65 °C for 12 h. The resulting precipitate was separated magnetically, washed severally with deionized water and dried; this product was denoted as MNP-NH_2_.

### Preparation of carboxylic functionalized superparamagnetic nanoparticles

**Figure 2:**
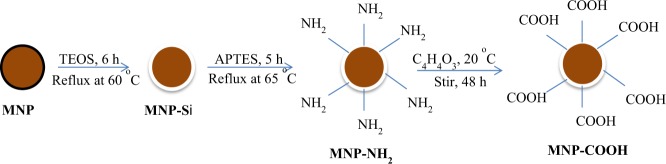
Synthesis route for the preparation of carboxylic acid functionalized superparamagnetic nanoparticles (MNP-COOH).

Carboxylic functionalized magnetic nanoparticles were prepared by adding previously dispersed MNP-NH_2_ (0.85 g) in dry DMF into a stirring solution of succinic anhydride (0.5 g) in dry DMF. The reaction was allowed to stir for 48 h before been separated magnetically, washed with ethanol and dried (Fig. 2)^32^. The product was denoted as MNP-COOH.

### Preparation of multiple nitrogen functionalized superparamagnetic nanoparticles

**Figure 3:**
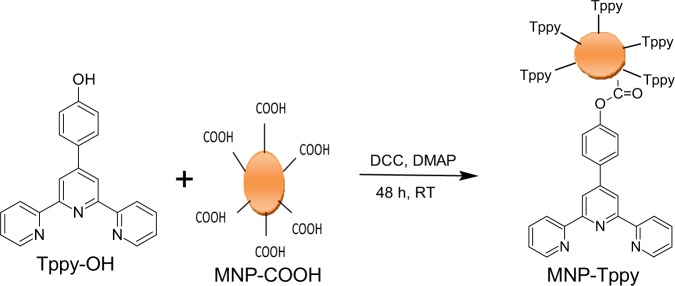
Synthesis route of terpyridine functionalized superparamagnetic nanoparticles (MNP-Tppy).

Covalent functionalization strategy was adopted in this study for the grafting of terpyridine ligand upon the surface of magnetic nanoparticles^33^. Carboxylic acid on the surface of carboxylic acid functionalized magnetic nanoparticles (MNP-COOH) 200 mg in 10 mL of dry DMF was activated with DCC (100 mg) with continous stirring in the dark for 24 h. Then Tppy-OH (200 mg) in 10 mL dry DMF was added slowly to the stirring solution in the presence of DMAP (50 mg), the suspension was allowed to stir for a further 24 h and separated magnetically, washed severally with deionized water and dried in a vaccum oven at 75 °C for 24 h. This final product was denoted as MNP-Tppy (Fig. 3).

### Preparation of adsorbate

Pure rhodamine-B standard powder was used for the preparation of adsorbate solution. Firstly, 1 g of pure rhodamine-B powder was dissolved in 100 mL deionized water in a 1000 mL volumetric flask. The solution was made up to mark in the volumetric flask using deionized water to obtain our standard solution. Adsorbate solutions employed for adsorption studies were obtained by diluting to the required concentrations and desired volume the standard solution.

### Procedure for adsorption experiments

Effect of pH, contact time, adsorbate concentration, temperature and adsorbent dose were investigated as part of the batch adsorption experiments. This was achieved in 100 mL glass bottles with screw caps on an orbital shaker at 150 rpm. The adsorbents were separated by magnetic means at the completion of each round of adsorption and the concentration of the adsorbate was determined by means of a UV-Visible spectrophotometer at 576.83 nm, the adsorption capacity was calculated by using equation (1). The effect of pH on the adsorption process was evaluated from pH values 2 to 10 by adjusting the initial solution pH to the desired pH by using 0.1 N HCl or NaOH solutions. Similarly, effect of time on the adsorption of Rh-B from aqueous solution was conducted from 0 to 240 min at three different absorbate concentrations while the effect of temperature was carried out at three different temperatures.

1 $${q}_{e}=(\frac{{C}_{o}-{C}_{f}}{W})\times \,V$$

Where *q*_*e*_ is the adsorption capacity in mg g^−1^, *C*_*o*_ and *C*_*f*_ is the initial and equilibrium rhodamine B concentration in ppm, *w* in mg is the amount of the adsorbent and *V* in mL is the volume of the rhodamine B used.

### Reusability experiment

Reusability experiment was conducted to ascertain the stable nature of the adsorbent. This was carried out by agitating 30 mg of desorbed adsorbent in 20 mL 50 ppm Rh-B solution for 2 h and the adsorption capacity was calculated by using equation 1. This process was repeated for 7 runs but before reusability test, a small amount of the spent adsorbent in 0.1 N HNO_3_ was agitated for 2 h using an orbital shaker to remove adsorbed Rh-B.

### Data analysis

Data were analyzed by means of the origin software statistical computing systems by considering an adjustment of the regression coefficient square (*R*^2^). For kinetic and isotherm studies, the model with highest *R*^2^ was considered best for describing the adsorption process. Similarly, spectral were obtained by plotting the data generated from the various instruments with the origin software^31^.

## Results and Discussion

### Characterization of materials

The effectiveness of the functionalized superparamagnetic nanoparticles for the removal of Rh-B from aqueous solution was evaluated by means of a batch adsorption experiment but prior to this; the characterization of this new material and ligand was carried out using a number of techniques so as to confirm the successful synthesis of this material.

#### Characterization of Tppy-OH

ESI Fig. 1 is the ^1^H NMR of 4-(2,2′:6′,2″-terpyridin-4′-yl)phenol, proton peaks clearly visible at δ: 8.80, 8.70, 8.05, 7.75, 7.80, 7.50, 6.95, 6.90 are ascribed to the presence of multiple nitrogen groups and aromatic proton resonance peaks in Tppy-OH. FTIR analysis revealed peaks at 3529 cm^−1^ (O–H str), 3056 cm^−1^ (aromatic C–H str), 1584 cm^−1^ (C=N str), 1467–1521 cm^−1^ (C=C str), 2794 cm^−1^ and 2879 cm^−1^ (indicating CH_2_ and CH groups respectively), 1259–1283 cm^−1^ (C–N str), 792 cm^−1^ for py-ring in-plane and 638 cm^−1^ out-of-plane deformation vibrations. This result compliments the ^1^H NMR result which reported the appearance of C=N peaks. Further confirmation of the synthesis of this product was done using ^13^C (ESI Fig. 2).

#### Characterization of nanoparticles

**Figure 4:**
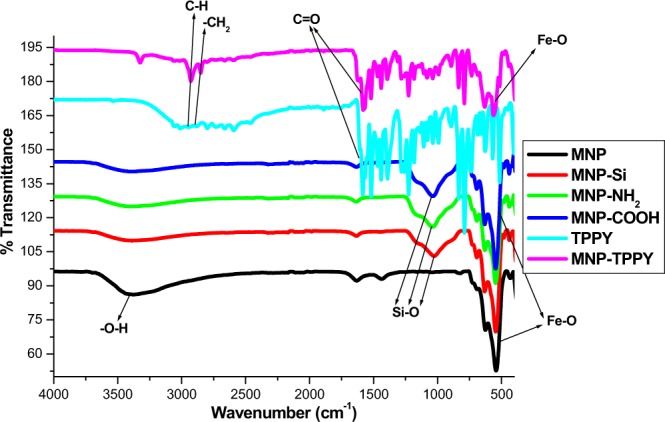
FTIR spectra of synthesized materials.

FTIR analysis: The FTIR spectra of MNP-Si, MNP-COOH and MNP-Tppy are presented in Fig. 4, the assignment of vibrational bands of the different functional groups observed in the samples were done. In Fig. 4, the synthesis of magnetic nanoparticles can be confirmed from the strong vibrational bands observed at about 520 cm^−1^ ascribed to the Fe–O bond of magnetic nanoparticles, the peak is sharp and intense which is a characteristic feature of this bond and in agreement with previously published studies^34,35^. Furthermore, the modification of the MNPs with TEOS for silica was responsible for the presence of a vibrational band around 1100 cm^−1^ attributed to Si–O bond. Similarly, Fig. 4 indicates that carboxylic acid group has been introduced on the surface of magnetic nanoparticles with the presence of vibrational bands around 1200 cm^−1^ and 1700 cm^−1^ assigned to C–O and C=O stretching frequencies respectively. The covalent functionalization of carboxylic acid functionalized magnetic nanoparticles with terpyridine ligand resulted in the formation of terpyridine functionalized magnetic nanoparticles (MNP-Tppy) with the observation of vibrational bands at about 1660 cm^−1^ for C=N and 1500 cm^−1^ for C–N stretching frequencies attributed to terpyridine ligands in addition to the vibrations present in the spectra of MNP-COOH confirmed our assertion that MNP-Tppy has been successful synthesized (Fig. 4c). Also, methyl and methylene groups were visibly observed in the spectra of MNP-Tppy at 2900 cm^−1^ and 2800 cm^−1^ due to symmetrical and asymmetrical vibrations of C–H from terpyridine ligand, report similar to this was reported by Chen *et al*.^34^. However, broad peaks around 3350 cm^−1^ in all the spectra could be assigned to O–H vibrational frequency from water molecules in the samples.

Surface properties: The BET analysis of MNP-COOH and MNP-Tppy is presented in Table 1. The surface area of MNP-Tppy was observed to increase upon covalent functionalization of MNP-COOH with Tppy-OH. Similarly, the pore volume of MNP-Tppy was observed to increase when compared to that of MNP-COOH. These results indicate that the level of functionalization increases the surface area and pore volume of adsorbents^36^. Both adsorbents can be seen to be mesoporous in nature since their pore diameter is less than 50 nm. This therefore means that the adsorption of Rh-B from aqueous solution will be greatly enhanced going by the moderately increased pore volume and large surface area of MNP-Tppy.

**Table 1:** Surface properties of synthesized MNP-COOH and MNP-Tppy.

Adsorbent	Surface area (m^2^/g)	Pore volume (cm^3^/g)	Pore diameter (nm)	pHpzc
MNP-COOH	84.4570	0.2766	12.7975	5.8
MNP-Tppy	94.8196	0.3197	13.4857	6.0

**Figure 5:**
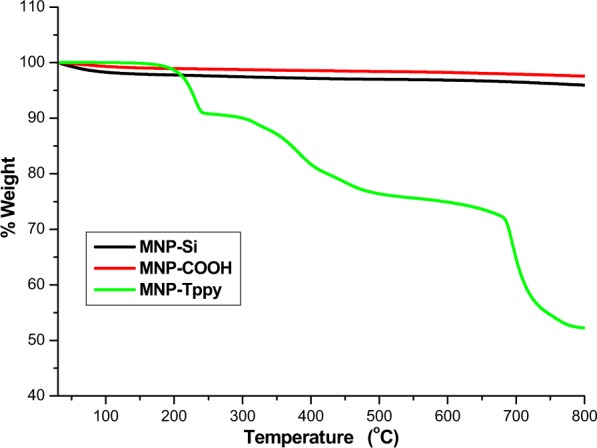
TGA curves of surface modified superparamagnetic nanoparticles.

Thermal analysis: Thermogravimetric analysis carried out from temperatures 30 °C to 850 °C for MNP, MNP-COOH and MNP-Tppy can be found in Fig. 5. It can be observed that MNP and MNP-COOH were to some extent thermally stable with little or no weight loss at temperatures above 450 °C. Contrastingly, significant weight loss was noticed in the thermogram of MNP-Tppy from 450 °C to 800 °C, this is attributed to the presence of Tppy-OH ligand. TEOS and APTES in the sample lose weights around 350 °C in all the thermogram while weight loss just about 450 °C is attributed to the presence of silicon carbon and carbon carbon bonds. In the thermogram of MNP-Tppy, further weight loss was observed after 450 °C which we attributed to the thermal decomposition of Tppy-OH ligand. This decomposition was not observed in the TGA curves of MNP and MNP-COOH showing that MNP was successful functionalized with Tppy.

**Figure 6:**
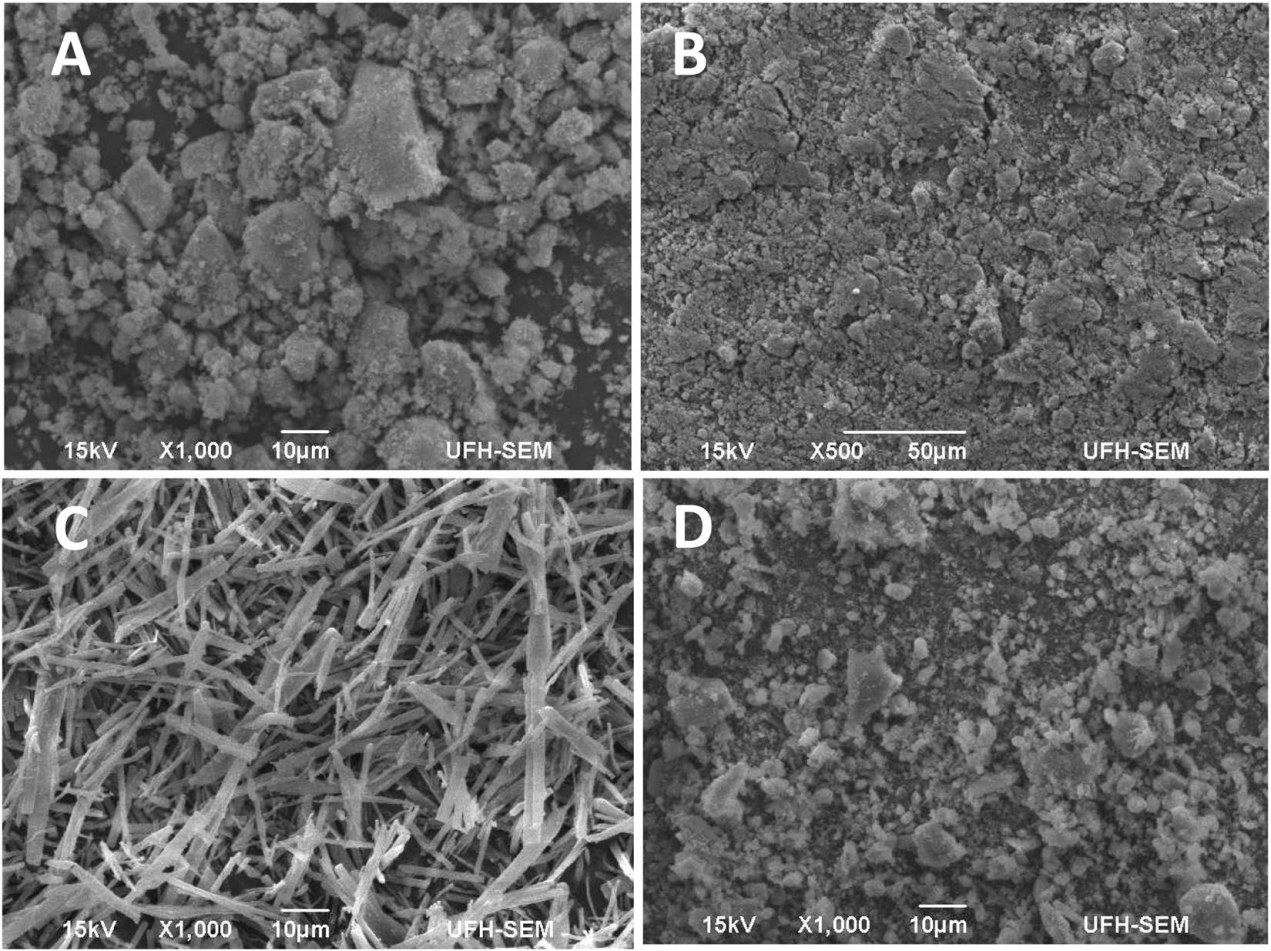
SEM images of (**A**) MNP-Si (**B**) MNP-COOH (**C**) Tppy-OH and (**D**) MNP-TPPY nanoparticles.

Microscopic analysis: SEM analysis of the synthesized modified magnetic nanoparticles and the ligand was carried out to enable us have an insight into the morphological and surface properties of the materials (Fig. 6). The SEM micrographs of Fig. 6(A) MNP-Si (B) MNP-COOH and (D) MNP-Tppy showed that all nanoparticles possess spherical shapes characteristics of magnetic nanoparticles. Interestingly, the images observed from SEM indicate that although agglomeration was observed, the spherical shapes of magnetic nanoparticles were still maintained even after functionalization. But as functionalization was steadily carried out on the magnetic nanoparticles from silica to terpyridine ligand, the observed agglomeration began to reduce which is in agreement with the reports already published in literature that surface modification helps to stabilize the magnetic core of magnetic nanoparticles^8,33^. The SEM image of Tppy-OH (Fig. 6C) indicates that a flower like material was synthesized which characteristics of amorphous materials of the nature of terpyridines. These observations are similar to results that have been recently reported on terpyridine ligands and their magnetic nanoparticles^37^.

**Figure 7:**
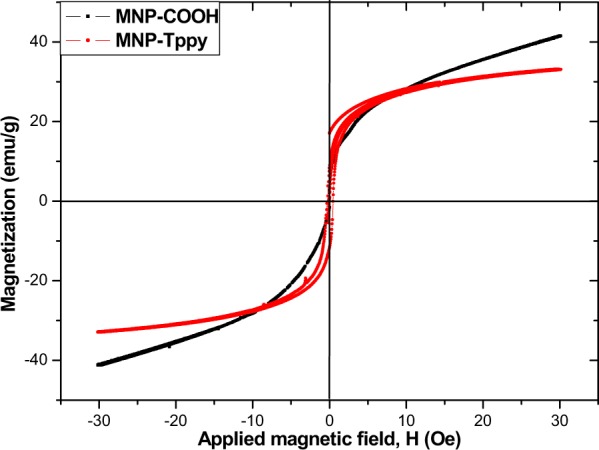
Magnetization plot of MNP-COOH and MNP-Tppy.

Superparamagnetic properties: Fig. 7 shows the magnetization measurement of MNP-COOH and MNP-Tppy. This measurement is important to assess the potential of separating the adsorbent by means of a magnet. The result of analysis shows that MNP-COOH and MNP-Tppy are superparamagnetic and showed no hysteresis at room temperature with magnetization value of a little above 30 emu/g and 40 emu/g respectively. Although, lower value of magnetization was obtained for MNP-Tppy compared to that of MNP-COOH which is ascribed to the presence of an additional modifier, Tppy-OH ligand on the surface of magnetic nanoparticles, the former still possesses high superparamagnetic property capable of enabling magnetic separation of the adsorbent with the aid of an external magnet.

### Adsorption experiments

To ascertain the efficiency of the adsorbent (MNP-Tppy) for the removal of Rh-B from aqueous solution, adsorption experiments were conducted by varying different parameters including pH, contact time, temperature, adsorbent amount and adsorbate concentration in order to arrive at the best experimental condition for the removal of this dye from solution.

#### Effect of pH

**Figure 8:**
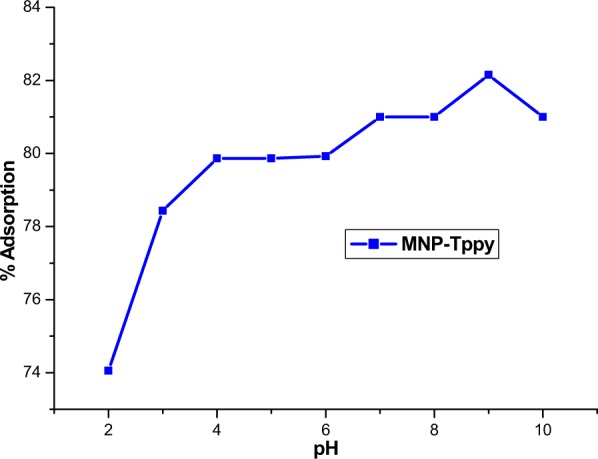
Influence of pH on the removal of Rh-B from aqueous solution. (conditions: 20 mL of 50 ppm Rh-B solution, 40 mg MNP-Tppy, 24 h agitation time, 20 °C and 150 rpm).

The effect of pH on the adsorption of Rh-B from aqueous solution was studied from pH range 2 to 10 at 22 °C, 150 rpm, 20 mL of 50 ppm adsorbate concentration and 40 mg MNP-Tppy (Fig. 8). The essence of varying the pH of Rh-B solution is because of the important role solution pH plays in adsorption processes as it is responsible for the charge density on the surface of MNP-Tppy as well as determines Rh-B ionization and speciation in aqueous solution. ESI Fig. 3 presents the plot of point of zero charge (pH_pzc_) of Rh-B and shows that the pH_pzc_ of Rh-B is about 6.0. A rise in the adsorption of Rh-B from aqueous solution was observed as the pH of the solution increases and remained constant at pH above 8 (Fig. 8), this is so because at pH < pH_pzc_, the surface of the MNP-Tppy remains positive and has affinity for negative ions. While in solution, the positively charged surface of MNP-Tppy competes with H^+^ ions for binding with negative ions which make it difficult to have a higher adsorption at these pH values. In contrast, pH > pH_pzc_ causes the surface of the adsorbent to be negative and favours the adsorption of positively charged Rh-B ions from aqueous solution. Although result of the effect of solution pH showed that adsorption of Rh-B was highest at pH 8, subsequent adsorption experiments were conducted at pH 6.0 in order to prevent the effect of electrostatic attraction at higher pH values.

#### Effect of time

**Figure 9:**
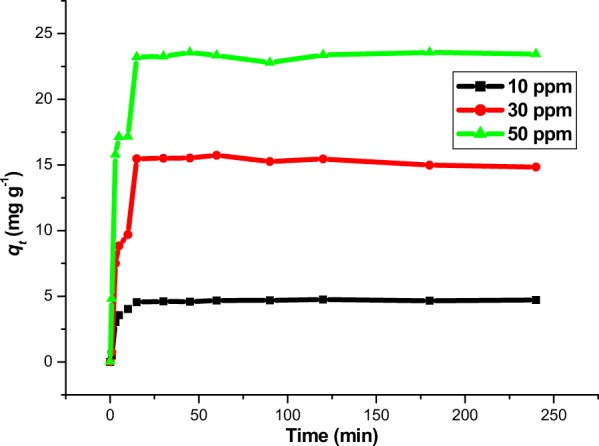
Effect of time for the removal of Rh-B from aqueous solution. (conditions: pH 6.0, 20 mL Rh-B solution, 30 mg MNP-Tppy, 20 °C and 150 rpm).

The effect of time on the adsorption of Rh-B from aqueous solution by MNP-Tppy was conducted as presented in Fig. 9. This study is necessary as effect of time is one of the major factors that determine adsorption efficiency. As shown in Fig. 9, a rapid Rh-B removal was noticed and the equilibrium time of adsorption was 15 min for different initial rhodamine B concentration. As the time was increased, it was observed that removal capacity remain stable and no distinct change was noticeable. For other adsorption studies, the contact time of 240 min was maintained in order to ensure that Rh-B was completely removed from solution. Increase in the adsorbate concentration also causes and increase in the adsorption capacity of MNP-Tppy (Fig. 9)^28,34^.

Kinetic Studies: For the possibility of adsorption process scale up, it is necessary to generate important data to evaluate the mechanism of the adsorption process^38^. The properties (physical and chemical) of the adsorbent and mass transfer are important parameters required for the evaluation of the mechanism of the adsorption process. In this study, we investigated the mechanism of the adsorption of Rh-B from aqueous solution onto MNP-Tppy by fitting the data obtained from the effect of time into four different kinetic models: pseudo first order, pseudo second order, elovich and intra-particle diffusion models. The equation and parameters of these models can be found in ESI Table 1. When the experimental data were fitted into the different kinetic models, the plots obtained (ESI Fig. 5) showed that pseudo-second order kinetic model can best describe the adsorption process owing to its highest *R*^2^ values. From Table 2, the value obtained for adsorption capacity in the experiment, *q*_*e*_ (exp.) is about the same as the one obtained for the calculated *q*_*e*_ values, (*q*_*e*_ (calc.)) across all three temperature range when the adsorption data was fitted to the pseudo second order kinetic model. This is a direct opposite of the values obtained for the three other models. Also, the rate was noticed to decrease as the initial concentration of Rh-B increases for pseudo second order compared to what was observed for the other three adsorption models. Based on these observations, we conclude that the adsorption of Rh-B onto MNP-Tppy can be best described by pseudo second order kinetic model. Therefore, the mechanism of adsorption in this process can be said to be bimolecular between the active sites on the surface of MNP-Tppy brought about by the multiple nitrogen atoms from the terpyridine ligand and the positively charged surface of Rh-B since pseudo second order kinetic model assumes that the interaction between an adsorbent and an adsorbate is bimolecular. Previously published work on the removal of Rh-B from aqueous solution also reported similar observation^39^.

**Table 2:** Kinetic parameters for the adsorption of Rh-B onto MNP-Tppy.

Kinetic models and parameters	Initial Rh-B concentration, *C*_*o*_ (ppm)		
	10 ppm	30 ppm	50 ppm
***q***_***e***_ **(exp.) (mg g** ^− **1**^ **)**	4.742	15.314	23.641
**Pseudo first-order model**			
*q*_*e*_ (calc.) (mg g^−1^)	1.565	10.231	3.473
*K*_*1*_ (min^−1^)	0.0482	0.1084	0.038
*R* ^2^	0.7519	0.7728	0.2729
**Pseudo second-order model**			
*q*_*e*_ (calc.) (mg g^−1^)	4.755	15.314	23.641
*K*_2_ (min^−1^)	0.1056	0.0209	0.0205
*R* ^2^	0.999	0.995	1.000
**Elovich model**			
*α* (mg g^−1^ min^−2^)	2059.17	270784.88	8991.18
*β* (g mg^−1^ min^−1^)	1.343	0.366	0.278
*R* ^2^	0.747	0.808	0.757
**Intra-particle diffusion model**			
*K*_*intra*_ (mg g^−1^ min^−1/2^)	0.228	0.842	1.110
*R* ^2^	0.470	0.524	0.493

#### Effect of adsorbent dose

**Figure 10:**
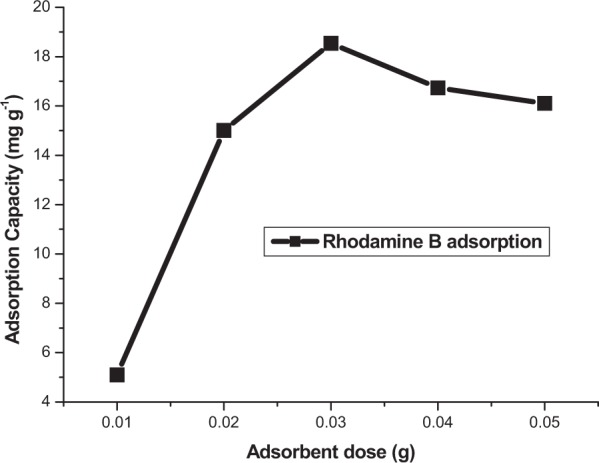
Effect of adsorbent dose for the removal of Rh-B from aqueous solution (conditions: pH 5.0, 150 rpm, 20 mL of 50 ppm Rh-B solution, 20 °C, 4 h).

The removal of Rh-B from aqueous solution by varying amounts of adsorbents is presented in Fig. 10. It can be observed that the higher the number of free sites on the adsorbent, the higher the adsorption capacity. The result obtained when the adsorbent dose was varied from 0.01 to 0.05 g indicates that maximum adsorption capacity (18.54 mg g^−1^) of Rh-B was noticed when the adsorbent dose was 0.03 g. This shows that adsorption increases as the number of active site increases until the surface of the adsorbent became saturated resulting to a gradual decrease in the adsorption capacity at adsorbent dose greater than 0.03 g. This adsorbent dose (0.03 g) where gradual decrease in the adsorption capacity was observed was adopted for further experiments in this study.

#### Effect of temperature

**Figure 11:**
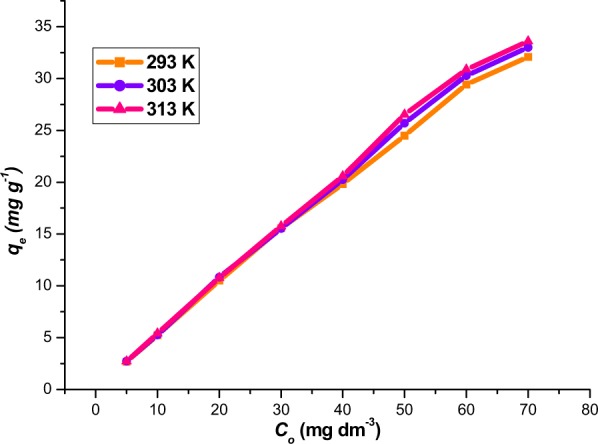
Influence of temperature on the removal of Rh-B from aqueous solution. (conditions: 20 mL Rh-B solution (50 ppm), 30 mg MNP-Tppy, 240 min agitation time and 150 rpm).

The influence of solution temperature on the adsorption of Rh-B from aqueous solution was studied at temperature range 20 °C to 40 °C at different initial concentration of the adsorbate (Fig. 11). The variation in the temperature of an adsorbate solution has been known to affect an adsorption process^40^. It can be observed that as the initial concentration of Rh-B increases, the removal capacity of MNP-Tppy also increases. Similarly, increase in temperature also causes an increase in the removal of Rh-B from aqueous solution; this is due to an increase in the diffusion of Rh-B ions in solution and a decrease in its viscosity which resulted in an increase in the adsorption capacity. It has been reported that increase in the temperature of adsorbate causes a higher rate in the diffusion of the solution^40^.

Isotherm study: The mechanism of adsorption and equilibrium process was determined by fitting the data obtained from the removal of Rh-B from aqueous solution by MNP-Tppy over the temperature range 20 °C to 40 °C into Langmuir and Freudlich equations (Equations 2 and 3 respectively).

2 $$(\frac{{C}_{f}}{{q}_{e}})=(\frac{1}{{q}_{m}\,b})+(\frac{{C}_{f}}{{q}_{m}})$$

3 $$\mathrm{log}\,{{\rm{q}}}_{{\rm{e}}}=\,\mathrm{log}\,{K}_{F}+(\frac{1}{n})\mathrm{log}\,{C}_{f}$$

where: *q*_*e*_ = adsorption capacity (mg g^−1^); *C*_*f*_ = concentration of adsorbate in solution at equilibrium (mg L^−1^); *q*_*m*_ = maximum monolayer capacity (mg g^−1^); b, Langmuir isotherm constant (L mg^−1^); K_F_, Freudlich isotherm constant (mg g^−1^)(L mg^−1^); n = adsorption intensity.

Isotherm study was conducted by computing the adsorption data obtained from the effect of temperature on the removal of Rh-B from aqueous solution by MNP-Tppy into two isotherm models. This study is important as it helps to determine the mechanism of adsorption process. Plots showing the fitting of these data into the two isotherm models are presented in ESI Fig. 6. Table 3, shows that Langmuir model best describes the process for the adsorption of Rh-B from aqueous solution by MNP-Tppy. This conclusion was made on the ground that the origin software^31^ employed for the linear square analysis of these data takes into consideration the highest coefficient of squares (*R*^2^). From this result (Table 3), it can be observed that Langmuir model has the highest *R*^2^ value across all temperatures. Also, the Langmuir constant, *q*_*m*_ for the adsorption of Rh-B onto MNP-Tppy increases from 72.46 to 113.64 mg g^−1^ as the temperature of the solution increases. Therefore, since *q*_*m*_ increased as solution temperature increases, we infer that this result is consistent and compliments the result we obtained for the effect of temperature where we observed that higher temperature favours adsorption capacity. Furthermore, the *N* values for the adsorption process are greater than 1 in the Freudlich model; this shows that the removal of Rh-B from aqueous solution by MNP-Tppy is practicable and realistic^41^.

**Table 3:** Isotherm values for the adsorption of Rhodamine B onto MNP-Tppy.

Adsorbate	Langmuir isotherm model			Freudlich isotherm model		
	*q*_*m*_ (mg g^−1^)	*b* (L mg^−1^)	*R* ^2^	*K*_*F*_	*n*	*R* ^2^
Rh-B	**20** **°C**					
	72.46	0.044	0.976	1.1687	1.220	0.993
	**30** **°C**					
	81.97	0.038	0.997	1.1841	1.213	0.993
	**40** **°C**					
	113.64	0.027	0.996	1.1872	1.203	0.993

In addition, a comparison of the adsorption capacity of Rh-B by MNP-Tppy obtained in this study with other data on Rh-B from literature (Table 4) indicates that this new adsorbent fare better for the removal of Rh-B from aqueous solution than previously published data on other adsorbents for Rh-B removal. We therefore infer that this adsorption process involving the removal of Rh-B from aqueous solution by MNP-Tppy shows a good performance as it is characterized by higher adsorbate removal as well as efficient magnetic separation.

**Table 4:** Comparison of Langmuir maximum adsorption capacity, *q*_*m*_ for Rh-B adsorption onto MNP-Tppy and other adsorbents.

Adsorbents	Conditions	*q*_*m*_ (mg g^−1^)	References
Palm kernel shell coated magnetic nanoparticle	pH 5, 200 mg L^−1^ adsorbate, 50 °C, 3 h.	625	^41^
Magnetic ZnFe_2_O_4_	pH 7, 25 mg L^−1^ adsorbate, 50 °C, 6 h.	12.1	^42^
MWCNT-COOH	pH 7.0, 100 mg L^−1^ adsorbate, 20 °C, 50 mg adsorbent 6 h.	42.68	^14^
CoFe_2_O_4_	pH 7.0, 100 mg L^−1^ adsorbate, 20 °C, 50 mg adsorbent 6 h.	5.17	^14^
Humic acid functionalized MNPs	pH 6.0, 50 mg L^−1^ adsorbate, 50 mg adsorbent, 15 min.	161.8	^43^
Fe_3_O_4_-MWCNT-COOH	pH 6.0, 15 mg L^−1^ adsorbate, 25 °C, 3 mg adsorbent, 1 h 20 min contact time.	11.44	^44^
Casuarina equisetifolia needles (Lignocellulose).	Unadjusted pH, 50 mg L^−1^ adsorbate, 25 °C, 5 mg adsorbent, 2 h.	82.34	^45^
Sodium montmorillonite	pH 7.0, 30 °C, 300 mg adsorbent 6 h.	42.19	^46^
MNP-Tppy	pH 5.0, 20 mL of 50 mg L^−1^ adsorbate, 40 °C, 30 mg adsorbent 6 h.	113.64	This study

Thermodynamic studies: Thermodynamic parameters namely: Gibbs free energy (ΔG°), entropy (ΔS°) and enthalpy (ΔH°) for the adsorption process at three different temperatures 293 K, 303 K and 313 K were calculated by using equations 4 and 5:

4 $${\rm{\Delta }}{{\boldsymbol{G}}}^{{\boldsymbol{o}}}=-\,RTlnK$$

5 $$lnk=-\,\frac{{\rm{\Delta }}{G}^{o}}{RT}=-\,\frac{{\rm{\Delta }}{H}^{o}}{RT}+{\frac{{\rm{\Delta }}S}{R}}^{{\rm{o}}}$$

where *R* is the gas constant (8.314 J mol^−1^ K^−1^), *T* is the absolute temperature in kelvin, ΔG° is the Gibbs free energy in kJ mol^−1^, K which is *q*_*e*_*/C*_*e*_ is the equilibrium constant at various temperatures, ∆S° in J mol^−1^ K^−1^ and ∆H° in kJ mol^−1^ are the intercept and slope of the graph of *ln k* against 1*/T* (ESI Fig. 7).

The values of these parameters for this adsorption process are presented in Table 5. Negative values were obtained for ΔG° for the adsorption of Rh-B by MNP-Tppy from aqueous solution and as temperature increases, ΔG° values decreases. These indicate the spontaneity of the process and that greater Rh-B adsorption is favoured by increase in temperature. Also, the huge randomness between the adsorbent and adsorbate during the adsorption process was noticed from the positive value obtained for ΔS°. Furthermore, positive ΔH° value obtained in this study suggests that the adsorption of Rh-B by MNP-Tppy is endothermic in nature. Liu and Liu (2008); Velickovic *et al*. (2013)^42,43^ reported that in order to understand the mechanism of adsorption by thermodynamic parameters, some conditions are required. For instance for an adsorption process with ΔG° and ΔH° values within (−80 and −400 kJ mol^−1^) and (80 and 200 kJ mol^−1^) respectively, the process is considered to be chemisorption while an adsorption process with ΔG^o^ and ΔH^o^ values within (20 and 0 kJ mol^−1^) and (21 and 20.9 kJ mol^−1^) respectively, the adsorption process is described as physisorption. From our result, the obtained ΔG^o^ and ΔH^o^ values is neither within the range of chemisorption nor physisorption processes indicating that the adsorption process for the removal of Rh-B by MNP-Tppy from aqueous solution was physico-chemical in nature.

**Table 5:** Thermodynamic parameters for the adsorption of Rh-B from aqueous solution by MNP-Tppy.

*ΔH*^*o*^ (kJ mol^−1^)	*ΔS*^*o*^ (J mol^−1^ K^−1^)	*ΔG*^*o*^ (kJ mol^−1^)			*R* ^2^
0.937	3.69	**293 K**	**303 K**	**313 K**	0.9869
		−1.220	−1.477	−1.836	

### Reusability studies

**Figure 12:**
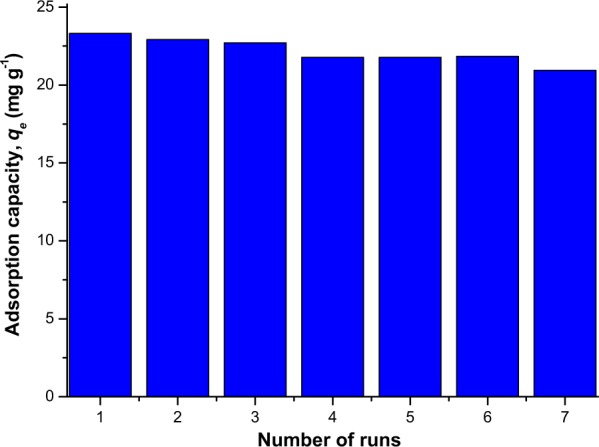
Reusability study involving the removal of Rh-B from aqueous solution after 7 runs.

The study to test for the ability of MNP-Tppy to be reused is presented in Fig. 12. This study is important to ascertain the stability of the adsorbent for practical use in the industry for cost reduction and prevention of the spread of secondary contaminant. This study was carried out by agitating at the completion of every adsorption process, a suspension of the used adsorbent in 0.1 molar HNO_3_ for 2 h. The desorbed adsorbent was now reused at seven different times for the removal of Rh-B from aqueous solution. The result of reusability indicates that the spent adsorbent can be reused without losing much of its adsorption capacity (Fig. 12); this implies that this adsorbent is stable and possesses good reusability ability and will enhance cost reduction for application in the industry.

## Conclusion

In this report, multiple nitrogen functionalities from terpyridine ligand were introduced onto the surface of magnetic nanoparticles via covalent linkage to enable negatively charged adsorbent for the removal of a positively charged Rh-B by electrostatic interaction. Characterization of the adsorbent showed that this novel adsorbent was successfully synthesized while batch adsorption experiment indicates that the removal of Rh-B by MNP-Tppy was pH dependent and followed pseudo-second order kinetic model. This study also shows that the removal of Rh-B from aqueous solution obeyed Langmuir isotherm model and the process was observed to be spontaneous, endothermic and realistic. Lastly the effectiveness of the reuse of this adsorbent was confirmed after seven runs with the adsorbent losing just about 3 mg g^−1^ of its adsorption capacity from its first use to its seventh use. Therefore, this adsorbent is efficient and stable with the capability to adequately remove dyes especially rhodamine B from wastewater generated from the industry.

## Supplementary information

Supplementary Information

## Data Availability

Data from this study will be made available on request.
