# Supplementary material for: Multiple nitrogen functionalized magnetic nanoparticles as an efficient adsorbent: synthesis, kinetics, isotherm and thermodynamic studies for the removal of rhodamine B from aqueous solution
Source: Sci Rep. 2019 Jul 4;9:9672. doi: 10.1038/s41598-019-45293-x (PMC6609594; doi:10.1038/s41598-019-45293-x)
Supplement: Supplementary file 1 — Supplementary Information [file 41598_2019_45293_MOESM1_ESM.docx]

**Multiple nitrogen functionalized magnetic nanoparticles as an efficient adsorbent: synthesis, kinetics, isotherm and thermodynamic studies for the removal of rhodamine B from aqueous solution**

Mike O. Ojemaye* and Anthony I. Okoh.

SAMRC; Microbial Water Quality Monitoring Centre, University of Fort Hare, South Africa.

Applied and Environmental Microbiology Research Group (AEMREG), University of Fort Hare, South Africa.

*Mike O. Ojemaye, E-mail: mojemaye@ufh.ac.za, Tel: +27603279482

**Scientific Reports**

Figure 1: ^1^H NMR of Tppy-OH ligand.

Figure 2: ^13^C NMR of Tppy-OH ligand.

Figure 3: pHpzc plot of MNP-Tppy.

Figure 4: Calibration curve of Rh-B at the different concentrations.

Table 1: Kinetic models investigated for the adsorption of Rh-B

| **Kinetics model** | **Equation** | **Parameters** |
| --- | --- | --- |
| Pseudo-first order | $\ln(q_{e}- q_{t})= \ln q_{e}-\frac{k_{1}}{2.303}t$ | *q_e,_ k_1_* |
| Pseudo-second order | $\frac{t}{q_{t}}= \frac{1}{k_{2}q_{e}^{2}}+ \frac{1}{q_{e}}t$ | *q_e_, k_2_* |
| Elovich | $q_{t}=\frac{1}{\beta} \ln\left( \alpha\beta\right)+\frac{1}{\beta}$ $\ln t$ | *α, β* |
| Intraparticle diffusion | $q_{t}=k_{id}\sqrt{t}+l$ | *k_id_, l* |

*q_t_*, amount of adsorbate adsorbed at time t (mg g^-1^); *q_e_*, amount of adsorbate adsorbed at equilibrium (mg g^-1^); *α*, adsorption rate constant (mg g^-1^ min^-1^); *β*, desorption rate constant (g mg^-1^); *k_1_*, pseudo-first order rate constant (min^-1^); *k_2_*, pseudo-second order rate constant (g mg^-1^ min^-1^); *k_id_*, intraparticle diffusion rate constant (mg g^-1^ min^0.5^); *l*= boundary layer thickness constant (mg g^-1^).

Figure 5: Kinetic plots of Rh-B adsorption onto MNP-Tppy.

Figure 6: Isotherm study for the removal of Rh-B from aqueous solution by MNP-Tppy.

Figure 7: Thermodynamic plot for the removal of Rh-B from aqueous solution.
